# Electrochemical impedimetric biosensors for food safety

**DOI:** 10.1007/s10068-020-00776-w

**Published:** 2020-07-10

**Authors:** Changhoon Chai, Se-Wook Oh

**Affiliations:** 1 Department of Applied Animal Science, Kangwon National University, Chuncheon, 24341 Republic of Korea; 2 Department of Food and Nutrition, Kookmin University, Seoul, 02707 Republic of Korea

**Keywords:** Electrochemical impedance spectroscopy, Biosensor, Food safety, Pathogen, Mycotoxin

## Abstract

Electrochemical impedimetric biosensors (EIBs) have a simple structure and can be used to rapidly and sensitively detect and measure hazards in food. EIBs detect and measure target molecules by transducing biochemical reactions on their surface to electrical signal outputs responding to a sinusoidal electrical signal input. Due to their structural simplicity and analytical sensitivity, EIBs are regarded as the most potent method of food hazard monitoring that can be implemented in the food supply chain. This paper discusses the theoretical background, structure, and construction of EIB and its applications in food safety.

## Introduction

Food safety is a key public health issue that begins with monitoring food hazards in including pathogens and chemical contaminants, and is achieved by eliminating or reducing food hazards to acceptable levels. As food hazards can enter the food supply chain at any point from farm to table, monitoring should be implemented at all points. Therefore, methods for monitoring food safety that can be easily implemented within the food supply chain are required. There have been marked advances in food safety monitoring technology over the past two decades, and various monitoring methods have been developed and are currently in use. In particular, electrochemical impedimetric biosensors (EIBs) have attracted a great deal of attention from food safety scientists and administrators. EIBs directly detect and measure target molecules with no sample preparation requirement, and can therefore be used for inline monitoring of hazards in the food supply chain. The sensitivity of EIBs for the detection and measurement of food hazards is comparable to or better than that of other biosensors and traditional methods (Ahmed et al., 2014; Bahadır and Sezgintürk, 2016; Malvano et al., 2019). EIBs can detect and measure food hazards in less than 1 h (Ahmed et al., 2014; Chai et al., 2010; Malvano et al., 2019). In combination with the Internet of Things (IoT), EIBs may evolve into real-time food safety monitoring systems (Durresi, 2016). Currently, the integration of EIBs into smart devices for food hazard detection has been intensively investigated (Huang et al., 2018; Rosati et al., 2019a). However, further research is required for incorporation of EIBs into the food supply chain. This review discusses the theoretical background, structure, and construction of EIBs, and their potential applications for food safety, to stimulate interest in their development for use in real-time inline food hazard monitoring.

## Theoretical background of EIBs

EIBs probe their target molecules by measuring impedance, which is enhanced by the formation of antibody–antigen or ligand–receptor complexes on their surface. Electrochemical impedance is the amount of opposition that an electrochemical cell (e.g., the EIB) presents to the flow of an electrical current on application of a small-amplitude sinusoidal voltage. The sinusoidal voltage input (*V*) as a function of time (*t*) can be expressed using the maximum amplitude of voltage (*V*_0_) and radial frequency (*ω*; *ω* = 2π*f*, where *f* is the linear frequency, represented by the number of cycles per second), and is also expressed as a complex number in Eq. 1:

1 $$ V = V_{0} \cdot \sin (wt) = V_{0} \cdot e^{jwt} $$

Current output (*I*) from EIB responding to the sinusoidal voltage input will be a sinusoid at the same *ω*, but shifted in phase (*ϕ*) and altered in terms of the maximum amplitude of the current (*I*_0_). Thus, *I* can be expressed as Eq. 2.

2 $$ I = I_{0} \cdot \sin (wt - f) = I_{0} \cdot e^{j(wt - f)} $$

According to Ohm’s law, impedance (*Z*) as a function of *ω* is *V* divided by *I* and can be represented as a complex number. Based on Euler’s relationship, *Z* can be expressed as a polar and rectangular coordinate form of a complex number, as shown in Eq. 3. *Z* in rectangular coordinate form can be characterized as a real part (*Z*_*re*_) and imaginary part (*Z*_*im*_), referred to as resistance and reactance, respectively. *Z*_*im*_ is enhanced due to *ϕ* and accounts for capacitance and inductance. However, the biological recognition elements and target molecules of EIBs, such as antibodies, antigens, receptors, DNAs, aptamers, etc., are not sufficiently electrochemically active to significantly alter the inductance (Rishpon and Buchner, 2005).

3 $$ Z = \frac{V}{I} = \frac{{V_{0} \cdot e^{j\omega t} }}{{I_{0} \cdot e^{j(\omega t - \phi )} }} = |Z| \cdot e^{j\phi } = |Z|(\cos \phi + j\sin \phi ) = Z_{re} + jZ_{im} $$

Electrolyte resistance (*R*_*s*_), double-layer capacitance (*C*_*dl*_), and charge-transfer resistance (*R*_*ct*_) at the electrode/electrolyte interface may be involved in the alteration of *Z* on application of a sample to EIBs. The electrolytes in a sample solution govern *R*_*s*_. *R*_*s*_ is independent of the target molecules in the sample solution, and can be determined by measuring *Z*_*re*_ of the sample solution at high *f*, from 0.1 to 10 MHz (Carminati et al., 2015; Itagaki et al., 2007; Manickam et al., 2012). *C*_*dl*_ depends on the thickness (*d*) of the electrical double layer (EDL) formed at the electrode/electrolyte interface, as well as the dielectric constant of the sample solution. The formation of antibody–antigen or ligand–receptor complexes on the EIB surface may alter the physicochemical characteristics of the interface between the EIB surface and sample solution, and may increase *d* in particular. If the effect of immunoreaction on the EIB surface on inductance is negligible, *C*_*dl*_ dominates *Z*_*im*_. *Z*_*im*_ is linearly related to the inverse of *C*_*dl*_ (Eq. 4), and *C*_*dl*_ is inversely proportional to *d* (Eq. 5). Thus, the formation of antibody–antigen or ligand–receptor complexes on the EIB surface decreases *C*_*dl*_ with increasing *d* (Carminati et al., 2015; Prodromidis, 2010). Changes in *C*_*dl*_ that are specific to immunoreactions on the EIB surface can be identified by measuring *Z* at *f* from 10 to 1000 Hz (Carminati et al., 2015; Prodromidis, 2010).

4 $$ Z_{im} \sim \frac{1}{{\omega C_{dl} }} $$

5 $$ C_{dl} \sim \frac{\varepsilon }{d} $$

where *ε* is the dielectric constant of the sample solution.

*R*_*ct*_ accounts for the diffusion of electrolytes from the bulk solution to the EIB surface, which is expected, especially when redox reactions occur (Carminati et al., 2015; Prodromidis 2010). Redox reactions can be enhanced by introducing redox probes, such as ferricyanide, into the sample solution or coupling redox reporters, such as graphene oxide, gold nanoparticle, and titanium carbide, with EIBs (Carminati et al., 2015; Li et al., 2017; Liang et al., 2019; Lu et al., 2012). Redox reactions affect current flow and *R*_*ct*_ (Carminati et al., 2015). With the formation of antibody–antigen, ligand–receptor, protein–aptamer, and DNA–DNA complexes on the EIB surface, *d* is increased and ions near the complexes are relocated, thereby altering *R*_*ct*_ (Bard, 1980; Manickam et al., 2012; Prodromidis, 2010). In particular, *R*_*ct*_ is altered more if the electrical potential of the EIB versus an additionally implemented reference electrode is maintained at a certain voltage (Bard, 1980; Park et al., 2018; Prodromidis. 2010). *R*_*ct*_ is an electrical parameter consisting of *Z*_*re*_, and the changes therein caused by immunoreactions on the EIB surface are frequency-dependent. *R*_*ct*_ can be characterized by *Z*_*re*_ at *f* from 0.1 to 1.0 Hz (Carminati et al., 2015; Prodromidis, 2010). Consequently, electrical parameters, including *R*_*s*_, *C*_*dl*_, and *R*_*ct*_, can be characterized by electrochemical impedance spectroscopy (EIS) of an EIB over a wide *f* from 0.1 Hz to 10 MHz (Maalouf et al., 2007a; Radhakrishnan et al., 2014).

**Fig. 1:**
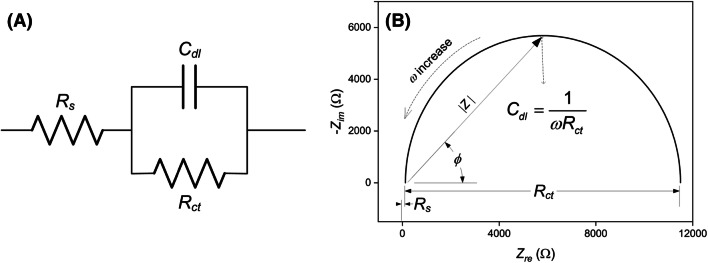
(**A**) A classical circuit model of the electrode/electrolyte interface. (**B**) Nyquist plot of the classical circuit model

The electrochemical impedance spectrum can be presented using Nyquist plots (− *Z*_*im*_ versus *Z*_*re*_) and Bode plots (*Z*, *ϕ*, *Z*_*re*_, and *Z*_*im*_ versus *f*). The electrical parameters of a circuit model equivalent to the EIB system can be characterized using a Nyquist plot. A classical circuit model with an electrode/electrolyte interface is presented in Fig. 1A. The Nyquist plot of the equivalent circuit in Fig. 1A is presented in Fig. 1B. On the Nyquist plot, *Z* is presented as a vector of length |*Z*|. The angle between the *Z* vector and the axis of *Z*_re_ is *ϕ *(Fig. 1B). The *Z* of the equivalent circuit in Fig. 1A can be expressed using *R*_*s*_, *R*_*ct*_, and *C*_*dl*_, and follows Eq. 6. With Eq. 6, the *Z*_*re*_ and *Z*_*im*_ can be expressed as Eqs. 7 and 8.

6 $$ Z = Z_{re} + jZ_{im} = R_{s} + \frac{1}{{\frac{1}{{R_{ct} }} + j\omega C_{dl} }} = \left[ {R_{s} + \frac{{R_{ct} }}{{1 + \omega^{2} C_{dl} R_{ct} }}} \right] - j\left[ {\frac{{\omega C_{dl} R_{ct}^{2} }}{{1 + \omega^{2} C_{dl}^{2} R_{ct}^{2} }}} \right] $$

7 $$ Z_{re} = R_{s} + \frac{{R_{ct} }}{{1 + \omega^{2} C_{dl}^{2} R_{ct}^{2} }} $$

8 $$ Z_{im} = - \frac{{\omega C_{dl} R_{ct}^{2} }}{{1 + \omega^{2} C_{dl}^{2} R_{ct}^{2} }} $$

As *ω* → 0 and ∞, limited forms of *Z*_*re*_ can be obtained as shown in Eq. 9. Thus, *R*_*ct*_ can be obtained by subtracting the minimum value of *Z*_*re*_ (*Z*_*re*_^*min*^) from the maximum value of *Z*_*re*_ (*Z*_*re*_^*max*^), as shown in Eq. 10.

9 $$ \omega \to 0,Z_{re} = R_{s} + R_{ct} \;{\text{and}}\;\omega \to \infty ,Z_{re} = R_{s} $$

10 $$ R_{ct} = Z_{re}^{\max } - Z_{re}^{\min } $$

The Nyquist plot of the equivalent circuit produces a semicircle with a radius of half *R*_*ct*_ (Bard, 1980). Hence, the maximum value of − *Z*_*im*_ (− *Z*_*im*_^*max*^) is centered at *Z*_*re*_ = *R*_*s*_ + *R*_*ct*_/2. Using Eq. 7, *C*_*dl*_ can be obtained with Eqs. 11 and 12.

11 $$ Z_{re} = R_{s} + \frac{{R_{ct} }}{{1 + \omega^{2} C_{dl}^{2} R_{ct}^{2} }} = R_{s} + \frac{{R_{ct} }}{2} $$

12 $$ C_{dl} = \frac{1}{{\omega R_{ct} }} $$

**Fig. 2:**
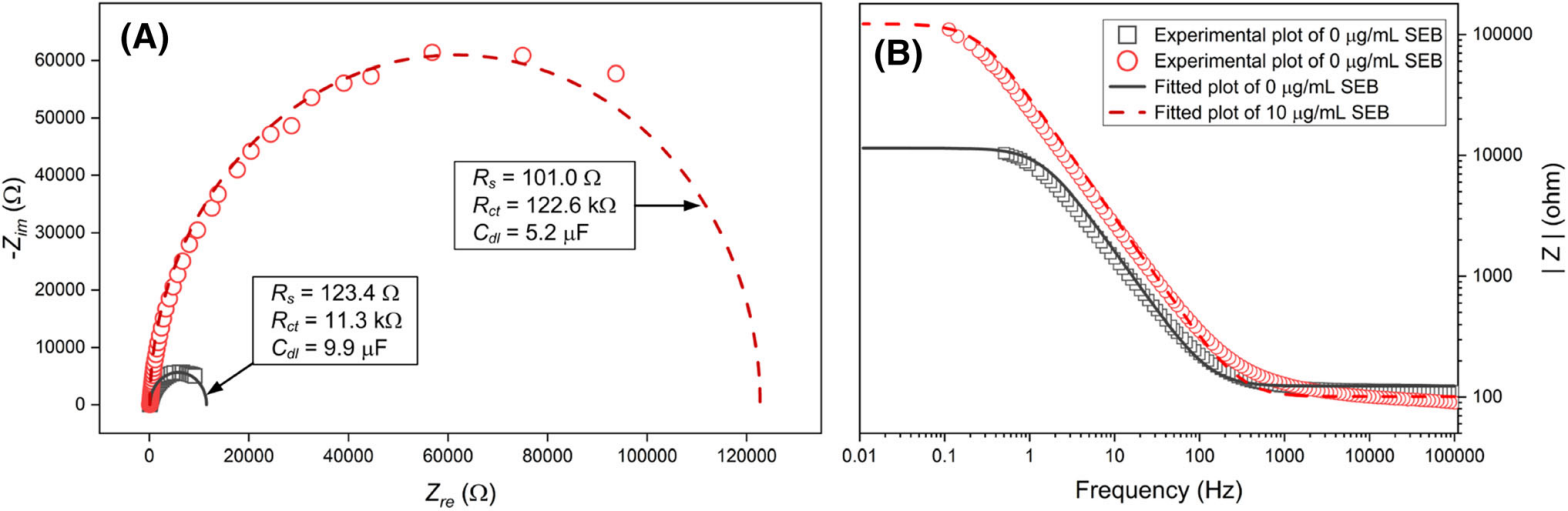
(**A**) Nyquist plots for an EIB for SEB, and mathematically fitted Nyquist plots. (**B**) Bode plots of |Z| versus *f* obtained from the EIB for SEB. EIB for SEB was developed using an anodic aluminum substrate and APTES. An anodic aluminum substrate with pores approximately 30 nm in diameter was treated with APTES. Anti-SEB was covalently immobilized on APES-SAMs deposited on the anodic aluminum substrate using glutaraldehyde. EIS of the EIB for SEB was performed at a biased potential of 0.1 V (vs. an Ag/AgCl reference electrode), in the absence or presence of 10 mg/mL SEB in 0.3% NaCl solution

However, experimentally obtained *Z*_*re*_ and − *Z*_*im*_ often do not produce a complete semicircle in a Nyquist plot due to the nonuniform current distribution on the electrode surface (Cheng and Chen, 2013). The Nyquist plot obtained by EIS measurement of EIBs must frequently be fitted. Figure 2A shows Nyquist plots obtained experimentally from EIS of the EIB for *Staphylococcus enterotoxin* B (SEB), and Nyquist plots fitted mathematically using EIS Spectrum Analyzer software v1.0 (Bondarenko and Ragoisha, 2005). The *R*_*s*_, *R*_*ct*_, and *C*_*dl*_ derived from the EIB for SEB were calculated based on the equivalent circuit presented in Fig. 1A. It is obvious that *R*_*ct*_ and *C*_*dl*_ derived from the EIB for SEB increased and decreased with complexation of SEB with anti-SEB antibodies immobilized on the EIB surface (Fig. 1C). As the *C*_*dl*_ decreased, *Z*_*im*_ also increased. Although a Nyquist plot is critical to characterize electrical parameters, *Z,* derived from an EIB, it is difficult to determine the dependence of the electrical parameters on the frequency. Bode plots provide frequency information, and are useful to determine the frequency range needed to obtain stable values of electrical parameters.

## Structure and construction of EIBs

**Fig. 3:**
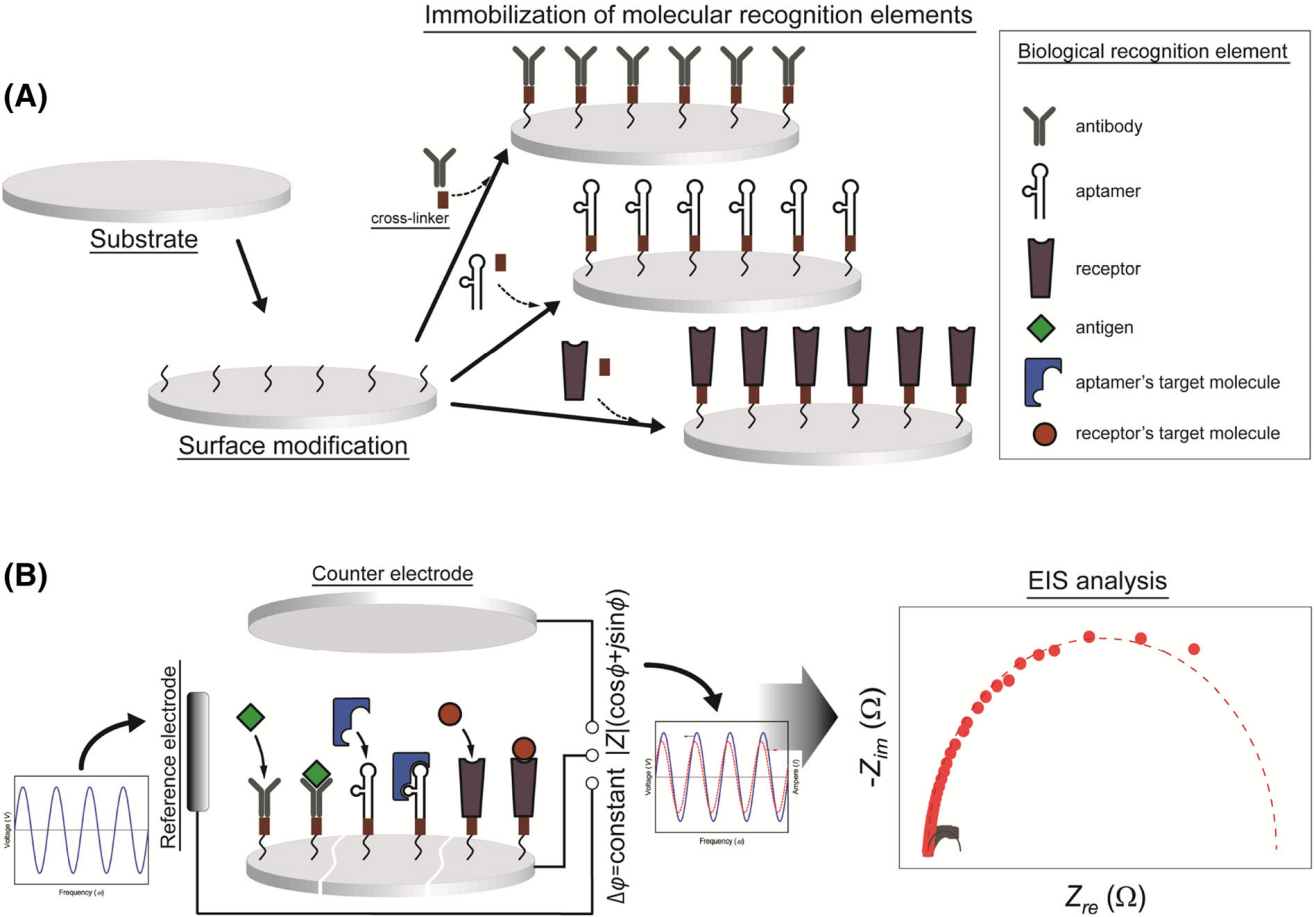
Schematic illustration of (**A**) the structure and construction, and (**B**) detection and measurement process, of EIB

An EIB consists of a signal transducer, an electrically conductive electrode substrate, and biological recognition elements (Fig. 3A) (Leca-Bouvier and Blum, 2005). For EIBs, an EIS analyzer acts as a signal transducer. An electrode substrate mediates biological recognition (Fig. 3B). Mercury, platinum, graphite, gold, stainless steel, silicon, and aluminum are the most frequently used materials for the electrode substrate (Săndulescu et al., 2015). There have been a number of studies on the use of nanoporous metal oxides and orderly structured carbon composites as the electrode substrate, to increase the surface area and sensitivity of EIBs (Ali et al., 2014; Ania et al., 2018; Bonanni et al., 2012; Chai and Takhistov, 2012). Metal nanoparticles have been used to intensify the electrochemical signal outputs from EIBs (Derkus et al., 2014; Lin et al., 2019; Peng et al., 2006).

Biological recognition elements include antibodies, aptamers, and receptors (Fig. 3A). To obtain impedimetric signal outputs specific for biological recognition at the EIB/sample solution interface, biological recognition elements should be immobilized intimately on the electrode substrate (Vashist et al., 2014). The electrode substrate may need to be chemically functionalized, and biological recognition elements can be chemically immobilized on the substrate by crosslinkers (Fig. 3A) (Nicosia and Huskens 2014; Vashist et al., 2014). Gold is reactive to thiols (Ron and Rubinstein, 1998). Thiol-based polymers, including peptides, proteins, and alkanethiols, can covalently bind to the gold surface and form self-assembled monolayers (SAMs) (Abad et al., 2006; Nicosia and Huskens 2014; Niu et al., 2012). Biological recognition elements can be immobilized by covalent binding with the SAMs of thiol-based polymers through the use of crosslinkers, such as protein A, protein G, and bifunctional amide compounds (Abad et al., 2006; Icoz et al., 2018). Metal oxides, such as silicon oxide and aluminum oxide, have hydroxyl groups on their surface and are reactive to silane compounds (Plueddemann, 1991). 3-Aminopropyltriethoxysilane (APTES) has been widely used to functionalize metal oxide surfaces (Chai et al., 2012a, b; Huy et al., 2011; Plueddemann 1991; Vashist et al., 2014). APTES bind electrostatically to metal oxides and form SAMs (Plueddemann, 1991). Antibodies and receptors can bind covalently to APTES with crosslinkers, such as glutaraldehyde (Fig. 3A) (Chai et al., 2012a, b; Vashist et al., 2014). APTES is also useful for functionalization of the surface of carbon composites (Luong et al., 2004; Zheng et al., 2013). Unlike the case of immobilization of proteins on the electrode substrate, DNA and aptamers must be conjugated with the thiol or amine group at the 3′ or 5′ end for immobilization on the electrode substrate (Lu et al., 2007; Paniel et al., 2013). Depending on the conjugated groups, DNA and aptamers can be immobilized directly on the gold surface or crosslinked with APTES-SAMs deposited on the electrode substrate (Keighley et al., 2008; Sauthier et al., 2002; Tam et al., 2009; Walsh et al., 2001; Wang et al., 2013).

## EIBs for detection of food hazards

Food hazard detection methods should not only be simple and easy to operate, thus allowing onsite monitoring, but also sensitive and reliable to prevent the consumption of contaminated and deteriorated foods. EIBs can identify biochemical reactions of biological recognition elements with their target molecules at the EIB/sample interface. Furthermore, EIBs do not require additional sample preparation steps, and are therefore among the most useful analytical methods for onsite detection of food hazards. This article discusses research regarding the use of EIBs for the detection of major food poisoning bacteria and mycotoxins.

Food poisoning bacteria are the most dangerous food hazards, and pose a major threat to human health. A large number of studies on the detection of food poisoning bacteria in foods have been conducted (Bridier, 2018; Hoorfar, 2011). Various antibodies that bind directly to food poisoning bacteria, such as pathogenic *Escherichia coli* and *Salmonella* spp., are commercially available, and EIBs can serve as a universal platform for these pathogens. An EIB with anti-*E. coli* O157:H7 on a gold-coated electrode detected the presence of 7 CFU/mL of *E. coli* O157:H7 in a ferrous solution (Joung et al., 2012). An increase in *R*_*ct*_ was observed as *E. coli* from a sample bound to anti-*E. coli* on the EIB, in proportion to the concentration of *E. coli* included in the sample (Joung et al., 2012; Maalouf et al., 2007b). The sensitivity of the EIB for *E. coli* O157:H7 was improved by attaching electron transferring mediators; the limit of detection (LOD) of the EIB was 3 CFU/mL (Malvano et al., 2018). Similar to the results of the EIB for *E. coli*, the binding of *Salmonella* spp. with anti-*Salmonella* immobilized on a gold electrode caused an increase in *R*_*ct*_ (Mantzila et al., 2008; Pournaras et al., 2008). The EIB for *Salmonella* spp., constructed on a gold electrode using tyramine as a surface modifier, exhibited a LOD of 20 CFU/mL *Salmonella* spp. (Liu et al., 2018). Aptamers, as biological recognition elements of EIBs, have been investigated due to their high binding specificity and affinity to their target bacteria (Teng et al., 2016). Aptamer-based EIBs for *E. coli* O157:H7 and *E. coli* O111 showed a LOD at the level of 100 CFU/mL (Brosel-Oliu et al., 2018; Luo et al., 2012). An aptamer-based EIB for *Salmonella* Typhimurium, constructed on a gold electrode functionalized by conductive polymer, could detect the presence of this bacterium at 3 CFU/mL (Sheikhzadeh et al., 2016). Viable *S.* Typhimurium could be selectively measured with an EIB developed using aptamers with high affinity to viable *S.* Typhimurium but poor affinity to dead *S.* Typhimurium (Labib et al., 2012). The EIB for viable *S.* Typhimurium had a LOD of 600 CFU/mL *S.* Typhimurium (Labib et al., 2012).

Mycotoxins are poisonous substances produced by fungi (Omotayo et al., 2019) that can cause disease and death in humans, and are therefore under strict governmental regulation (European Commission, 2010; KFDA, 2020; US FDA, 2016). The development of EIBs for mycotoxins has focused on the detection of ochratoxin A and aflatoxins, due to their prevalence and toxicity (Malvano et al., 2019; Omotayo et al., 2019). An EIB with anti-ochratoxin A immobilized on an indium oxide electrode showed a linear response, in terms of *R*_*ct*_, to ochratoxin concentrations ranging from 1 to 10 ng/mL (Khan and Dhayal 2009). An EIB for ochratoxin A built on a gold electrode showed similar results to one based on an indium oxide electrode (Radi et al., 2009). The acceptable limit established for ochratoxin A in food products is 5 ng/g (Codex STAN 1995). The sensitivity of the EIBs described above was not sufficient to meet existing regulations established for ochratoxin A. An EIB that could measure ochratoxin A at concentrations in food products below 0.5 ng/g was reported (Tang et al., 2016). That EIB, based on competitive immunoreaction, had a reference ochratoxin A-immobilized carbon electrode and signal tags (anti-ochratoxin A-immobilized and manganese oxide-adsorbed graphene oxide nanosheets), and measured impedance; signal tags bound to the electrode could detect the presence of 0.055 pg/mL ochratoxin A (Tang et al., 2016).

Aflatoxins are a family of mycotoxins mainly produced by *Aspergillus* species (Dutton et al., 1985). Four major types of aflatoxins are found in food: aflatoxin B_1_, B_2_, G_1_, and G_2_ (Bennett and Klich, 2003). Aflatoxin B_1_ is the most common and toxic aflatoxin in food, but all aflatoxins are toxic and carcinogenic (Wakenell, 2016). Aflatoxin regulations are often based on the sum of aflatoxin B_1_, B_2_, G_1_, and G_2_, and the maximum permissible level of total aflatoxins in food established by the CODEX Alimentarius Commission is 15 ng/g (Codex STAN 1995). Antibodies specific to aflatoxin B_1_ are commercially available, and many antibodies developed using aflatoxin B_1_ show good cross-affinity to aflatoxin B_2_, G_1_, and G_2_ (Ertekin et al., 2016; Gathumbi et al., 2001). An EIB with anti-aflatoxin B_1_ immobilized on the carbon electrode, where carbon nanotubes were physically adsorbed, showed a linear increase in *R*_*ct*_ with increasing level of aflatoxin B_1_ from 0.1 to 10 ng/mL (Yu et al., 2015). A highly sensitive EIB that could directly measure aflatoxin B_1_ was developed by immobilizing anti-aflatoxin B_1_ on carbon nanotubes covalently anchored on the gold electrode. The carbon nanotubes were covalently anchored on the surface of a gold electrode via formation of cysteine SAMs on the gold surface and subsequent activation of cysteine SAMs using 1-ethyl-3-(3-dimethylaminopropyl)carbodiimide (EDC) and *N*-hydroxysuccinimide (NHS). The EIB showed a linear response, in terms of *R*_*ct*_, to aflatoxin B_1_ concentrations ranging from 0.1 to 20 pg/mL (Costa et al., 2017). The sensitivity of the EIB for aflatoxin B_1_ was improved by graphene oxide and conductive polymer (Wang et al., 2015). Graphene oxide was deposited on a carbon electrode and anti-aflatoxin B_1_ was cross-linked to the graphene oxide with conductive polymer. The EIB for aflatoxin B_1_ developed using graphene oxide and conductive polymer exhibited a significant increase in *R*_*ct*_ even in the presence of 10 fg/mL aflatoxin B_1_. The EIB also showed a linear increase in *R*_*ct*_ with increasing aflatoxin B_1_ concentration from 10 fg/mL to 10 pg/mL (Wang et al., 2015). A cost-effective, disposable but highly sensitive EIB for aflatoxin B_1_ was developed using a gold CD-trode (the gold layer used for recordable compact discs) (Foguel et al., 2016). Anti-aflatoxin B_1_ was immobilized covalently on a gold CD-trode by surface functionalization, using lipoic acid and subsequent EDC/NHS activation. The *R*_*ct*_ from the EIB increased in proportion to the increase aflatoxin B_1_ concentration, from 1.56 to 31.2 ng/mL, and had a LOD of 0.11 ng/mL.

In conclusion, EIBs have a number of advantages over conventional and optical biosensors. Unlike optical-based biosensors, EIBs do not require excitation sources, filters, or lenses. EIBs can directly qualify and quantify their target molecules in food, and have comparable or better sensitivity than optical biosensors. The EIB is a versatile platform that can be modified to measure different food hazards through replacement of biological recognition elements. EIBs can be manufactured using consumer-grade inkjet printers (Rosati et al., 2019a, b). The EIB for a bacteriophage produced using an inkjet printer showed better sensitivity than a traditional method for bacteriophage detection (Rosati et al., 2019a). EIBs appear to be a sensitive and cost-effective means of food hazard detection suitable for mass production. With advances in mobile phone technology, there have been a number of studies concerned with integration of EIBs into smartphones (Huang et al., 2018; Rosati et al., 2019b). In the near future, EIBs are expected to be implemented throughout the food supply chain as for inline and real-time monitoring of food hazards.

## List of symbols

*V*: The sinusoidal voltage input
*V*_0_: The maximum amplitude of *V*
*I*: The current output
*I*_0_: The maximum amplitude of *I*
*f*: The linear frequency
*t*: Time
*ω*: The radial frequency
*ϕ*: The phase shift of *I*
*Z*: The impedance
|*Z*|: The absolute value of *Z*
*Z*_*re*_: The real part of *Z*
*Z*_*im*_: The imaginary part of *Z*
*Z*_*re*_^*min*^: The minimum value of *Z*_*re*_
*Z*_*re*_^*max*^: The maximum value of *Z*_*re*_
*R*_*s*_: The electrolyte resistance
*C*_*dl*_: The double-layer capacitance
*R*_*ct*_: The charge-transfer resistance
*ε*: The dielectric constant
*d*: The thickness of the electrical double layer
