# Correction to: Electrochemical impedimetric biosensors for food safety

**DOI:** 10.1007/s10068-020-00808-5

**Published:** 2020-09-05

**Authors:** Changhoon Chai, Se-Wook Oh

**Affiliations:** 1 Department of Applied Animal Science, Kangwon National University, Chuncheon, 24341 Republic of Korea; 2 Department of Food and Nutrition, Kookmin University, Seoul, 02707 Republic of Korea

## Correction to: Food Sci Biotechnol (2020) 29(7):879–887 10.1007/s10068-020-00776-w

The article “Electrochemical impedimetric biosensors for food safety”, written by Changhoon Chai and Se-Wook Oh, was originally published Online First without Open Access. After publication in volume 29, issue 7, page 879–887 the author decided to opt for Open Choice and to make the article an Open Access publication. Therefore, the copyright of the article has been changed to © The Author(s) 2020 and the article is forthwith distributed under the terms of the Creative Commons Attribution 4.0 International License (http://creativecommons.org/licenses/by/4.0/), which permits use, duplication, adaptation, distribution and reproduction in any medium or format, as long as you give appropriate credit to the original author(s) and the source, provide a link to the Creative Commons license, and indicate if changes were made.

The original article has been corrected.

